# Implementation research design: integrating participatory action research into randomized controlled trials

**DOI:** 10.1186/1748-5908-4-69

**Published:** 2009-10-23

**Authors:** Luci K Leykum, Jacqueline A Pugh, Holly J Lanham, Joel Harmon, Reuben R McDaniel

**Affiliations:** 1 VERDICT, a VA HSR&D REAP at the South Texas Veterans Health Care System, San Antonio, Texas, USA; 2 Department of Medicine, School of Medicine, University of Texas Health Science Center at San Antonio, San Antonio, Texas, USA; 3 School of Business, Fairleigh Dickinson University, Madison, New Jersey, USA; 4 Department of Information, Risk and Operations Management, McCombs School of Business, The University of Texas at Austin, Austin, Texas, USA

## Abstract

**Background:**

A gap continues to exist between what is known to be effective and what is actually delivered in the usual course of medical care. The goal of implementation research is to reduce this gap. However, a tension exists between the need to obtain generalizeable knowledge through implementation trials, and the inherent differences between healthcare organizations that make standard interventional approaches less likely to succeed. The purpose of this paper is to explore the integration of participatory action research and randomized controlled trial (RCT) study designs to suggest a new approach for studying interventions in healthcare settings.

**Discussion:**

We summarize key elements of participatory action research, with particular attention to its collaborative, reflective approach. Elements of participatory action research and RCT study designs are discussed and contrasted, with a complex adaptive systems approach used to frame their integration.

**Summary:**

The integration of participatory action research and RCT design results in a new approach that reflects not only the complex nature of healthcare organizations, but also the need to obtain generalizeable knowledge regarding the implementation process.

## Background

A gap exists between what is known to be effective and what is actually delivered in the course of usual medical care in international health systems [1-5]. The aim of implementation research is to reduce this gap through identifying methods to improve clinical practice in a generalizeable way. Implementation research tries to understand how an intervention designed to improve clinical practice and tested in a limited, controlled setting can be implemented across a wide range of settings. These implementation research efforts have ranged from interventions focusing on individual provider behavior, to those with a more general educational focus, to those designed to address specific barriers to change, but these efforts share in common only small to modest effects on outcomes [6-10].

Interventions that are multi-pronged in approach, or that target organizations rather than individuals, may be more likely to be successful [11-13]. However, these may also be more difficult to translate from one institution or setting to another because of inherent differences between institutions. These differences arise because healthcare organizations are not static, but are constantly adapting and evolving in response to changes in their local environments, making one-size-fits-all interventions that attempt to reduce local variation less likely to be successful.

This leads to a profound dilemma in implementation research: how do we design interventional trials that are generalizeable, but also have enough flexibility to be meaningful and more likely to be successful locally? To put this another way, how can we marry what many consider to be the ideal of the randomized controlled trial (RCT) with methods that address the difficulty of retaining interventional fidelity across institutions, and also address the more individualized, institutional needs of institutions when it comes to actually making an intervention work on a local level? The goal of this paper is to explore the integration of participatory action research (PAR) with a RCT study design as a mechanism for informing and improving our ability to translate research findings into general practice.

## Why there is a need to consider different research methods in healthcare organizations

A growing literature suggests that healthcare organizations are complex adaptive systems (CAS) [13-19]. CAS are comprised of individuals who learn, inter-relate, and self-organize to complete tasks. They also co-evolve with their environment, responding to external forces in ways that in turn reshape their external environment. Most importantly, CAS are characterized by non-linear interactions that may lead to outputs, or 'emergent properties,' that are not entirely predictable.

Conceptualizing healthcare organizations as CAS has important implications for how we think about intervening in such systems, as the CAS framework reinforces the idea that each system is unique, and that interventions cannot easily be moved from one organization to the next with predictable results [13,17,20,21]. The CAS framework goes further, however, by suggesting that it is only through leveraging each system's pattern of interconnections between individuals that interventions will be optimally effective. Thus, to have the biggest impact, it is necessary to not only take into account differences between systems, but to exploit these in a way that will lead to maximal results. The implication is that the local participants will have the greatest ability to accomplish this.

The idea of performing a RCT in CAS requires us to rethink several key points about RCTs. First, the notion that a single intervention can be applied in a standardized way is not applicable. Therefore, we need to pay attention to what elements of an intervention could or should be common to all sites, and what can be varied locally. Second, the CAS framework should lead us to rethink the idea of monitoring fixed 'endpoints' at certain pre-specified points in time. Instead, we must pay attention to the implementation of an intervention throughout time, to how the intervention impacts the interdependencies within the system, and to the potentially unpredictable impacts of interventions. This requires a different level of monitoring, one that can best be done by local participants. Finally, the application of CAS to clinical systems encourages the idea that the intervention itself will evolve over time as the organization in which it is implemented changes. This may make the intervention more or less effective over time.

Thus, reconceptualizing clinical and healthcare organizations as CAS makes new approaches to implementation research necessary. A way of not only accounting for but taking advantage of local differences in healthcare systems is needed, but needs to be balanced by a research design framework that allows for some level of generalizability. The CAS nature of healthcare systems may make the PAR approach a particularly appropriate one for use in healthcare. PAR recognizes the importance of relationships, feedback loops, and the ability of participants to self-organize within a dynamic system -- three hallmarks of CAS.

## Participatory action research defined

PAR is a technique derived over the last 40 years from the sociological, organizational, educational, and evaluation research literatures [22-24]. It is a design that partners the researcher and participants in a collaborative effort to address issues in specific systems. It is a collaborative, cyclical, reflective inquiry design that focuses on problem solving, improving work practices, and on understanding the effect of the research or intervention as part of the research process. It explicitly calls for making sense of the impact of change, and refining actions based on this impact. Essential elements and typical methods of action research are shown in Table 1[22-56], derived from reviewing definitions of PAR across disciplines and qualitatively analyzing these definitions for themes and commonalities.

**Table 1:** Essential elements of participatory action research

**Quotes from Published Definitions**	**References**
**Names Used**	

**Participatory action research** Qualifiers: cooperative inquiry; appreciative inquiry; community-based participatory research; action learning; action science; developmental action inquiry	[22-24,35-40,44-50]

**Purpose of the Action Research**	

**Generation of new knowledge ** Qualifiers: practice-grounded, compelling enough to motivate to action; answer a question of importance to each other	[24,32,34,36,38,39,46,51,52]

**Change** Qualifiers: social change; improvement; improve health/well-being; take action; solution generation; planning action steps; engage in quest for information/ideas to guide future actions	[22-24,32,35-37,52,53]

**Educating**	[23,24,32,36,54]

**Theory generation or refinement**	[23,52]

**Relationship building** Qualifiers: strengthen relationships among group members, learn to integrate individualizing characteristics with a deeper communion with others and the world; involvement;	[23,27,38,55]

**Developmental/Transformative for the individuals or organizations involved** Qualifiers: a re-educative process that develops capabilities and transforms individuals/teams through experiential engagement; empowerment; reciprocal transfer of expertise	[23,24,35,51,53,54]

**Methods**	

**Problem-focused** Qualifiers: problem identification; diagnosing a problem; define a pressing problem; an agreed area of human activity; solution generation; planning action steps; engage actively in the quest for information and ideas to guide future actions	[22,23,34-39,54]

**Systematic**	[32,36]

**Cyclical** Qualifiers: emergence; adaptive cycles of action-feedback-action-feedback-action; repeated episodes of reflection and action; between meetings, members inquire into their own practice, observe, and implement new actions to help learn something new about the question; four phases of reflection and action; experimentation; learning at each step to inform the next set of decisions/actions; evaluation leads to diagnosing the situation anew based on incremental learnings	[23,34,35,37,39,51]

**Reflective** Qualifiers: self-reflective; members reflect together on their work; inquiring deeply into assumptions and root causes, and transferring learning at multiple levels	[23,27,38-40,52]

**Collaborative Design and Evaluation** Qualifiers: partnership; collective; group activity; mutualistic; inclusive; collaboration shapes and transforms methods; co-learning; participation of all relevant constituencies or stakeholders; involve all participants in all aspects of the research process; organization members participate throughout the research process from the initial design to the final presentation of results and discussion of their implications; reciprocal transfer of expertise; shared decision making power; mutual ownership of the processes and products of the research enterprise; facilitators and group participants co-author reports to present findings; participate in the research processes, which in turn are applied in ways that benefit all participants; multiple person, multiple perspective with participants as co-researchers	[22-24,32-39,49,51,52]

**Context specific** Qualifiers: Must be applicable to the system in which the inquiry takes place	[23,46,51,56]

**Studying the whole or the patterns rather than the parts**	[33,35,52]

**Qualitative and quantitative data collection and analysis** Qualifiers: mixed method designs collecting/analyzing both qualitative and quantitative data in single study; concurrent triangulation with multi-strand, multi-wave design; data collected/analyzed simultaneously/iteratively	[23,28,34,52]

**Who**	

**Researchers** Qualifiers: Professional action researchers, core research team members, researchers	[22-24,38,49]

**Whoever is affected by the problem being studied** Qualifiers: Requisite variety; system members; communities; those affected by the issue being studied; representatives of organizations; members of an organization or community seeking to improve their situation; group of peers	[24,32,38,39,46,49]

**Fields Represented**	

**Health Related**: Public Health, Primary Care, Patient Care, Nursing, Health Education, Health Sociology, Disability Research, Environmental Health, Injury Research, Mental Health, Reproductive Health	
**Non-Health Related**: Anthropology, Business Administration (Organizational Change/Development, Management, Human-Information System Interfaces), Sociology, Community Development, Community Psychology	

PAR has been influential in healthcare literature. Two systematic reviews of what may be considered PAR in healthcare settings are available. The UK National Health Service funded a systematic review of action research, published in 2001 [23]. 'Initiatives that persisted at the same location were found in 32 studies (54%) and, in a small number (four studies, 13%), an effect beyond their location was claimed.' In 2004, the Agency for Healthcare Research and Quality sponsored an evidence report on community-based participatory research [24]. This review found only 12 completed interventional studies, four of which were RCT's. Findings revealed modest positive health outcome findings, but the reviewers could not determine whether this benefit could be attributed to the community-based participatory research methods. Both reviews suggest the need to further understand what constitutes high-quality PAR and how best to evaluate the quality and outcomes of such research. More recently, a number of studies have been published using PAR to approach a variety of healthcare issues, including physical activity and obesity in young people [25,26], health disparities [27], hypertension and diabetes management [28], primary care delivery [29], and disaster planning [30].

PAR shares concepts with both action research and participatory research, but is not identical to these approaches.

## Similarities and differences between PAR and quality improvement strategies

While the term 'PAR' is not widely used in clinical circles, many continuous quality improvement (CQI) techniques, such as Deming's total quality improvement, Six Sigma techniques, and the Institute for Healthcare Improvement's learning collaboratives, have features that are consistent with PAR. First, they call for involvement of a team of key individuals, particularly those with a fundamental knowledge of the context and need for improvement, to be involved in the process. Second, they call for focusing a team around a specific problem. Third, they involve a cyclical approach with repeated cycles of incremental improvement, analogous to 'plan-do-study-act.' Finally, both PAR and CQI are meant to be transformative for the individuals involved, so that they have the skills to problem solve in new scenarios.

An important difference between PAR and CQI is that the latter typically assumes a reductionist system that can be improved by looking at specific steps in healthcare processes. PAR's emphasis on the relationships between individuals in the system, and their ability to self-organize over time, implies an inherent applicability to CAS. An additional difference between PAR and CQI approaches is that the primary goal of the latter is to do an intervention, while that of the former is also to learn something about the implementation process itself.

## How PAR may be integrated with randomized controlled trails in implementation research design

We propose integrating the RCT and PAR approaches to retain the 'rigor' of the RCT with the local sensibility brought by PAR. This integration informs several elements of a combined design: the intervention, the endpoints, and the process of measurement. Table 2 summarizes key elements of PAR and RCT, and how these specific elements may be incorporated into an integrated PAR/RCT approach.

**Table 2:** Elements of PAR, RCT's, and integrated PAR/RCT

**PAR**	**RCT**	**Integrated PAR/RCT**	**Example of PAR/RCT**
Collaborative design	Externally created, standardized interventions	Key elements of intervention are locally implemented based on collaborative discussion	Use of site PIs in each unique study site as collaborators with study PIs in intervention design

Internal control	External control	Joint control	Site PIs with local or shared authority

Local applicability	Generalizeable	Use local findings to inform universal understanding	Consider local insights gleaned from the implementation process as data that will form the basis for a general understanding

Acknowledge unique local environments	Uniqueness minimized through random assignment	Incorporation of local conditions into overarching approaches	Address local barriers in intervention implementation

Reveal biases	Reduce bias	Use bias to form basis of generalizeable understanding	Allowing bias into the design may lead to a better understanding of the implementation process.

Reflective process throughout intervention	Endpoints/measurement set in advance	Time function or endpoints may vary within boundaries Reflection both within and across sites	Modify endpoints based on results Incorporate reflection periods into study design.

No comparisons, internal focus	Comparisons between arms	Comparisons based on 'content analysis' of internal understandings and lessons	Use of qualitative methods to probe themes from implementation experiences between sites

To integrate PAR into an RCT framework, we will need to move away from the proscribed interventions of the 'traditional' RCT in favor of locally designed interventions that meet a general goal or strategy. Elements of PAR may be important additions to intervention design in implementation research, particularly the need for local input into intervention design, and the need for sites to continue to change over the course of an intervention based on the success of the intervention. PAR may help us to focus less on the medical content of the intervention and more on the processes of group facilitation, reflection, and relationship building that may be the more generalizeable components of the intervention. These activities should be made explicit elements of an intervention to allow for the incorporation of local conditions or context into the research design.

Non-healthcare literatures suggest that participation and decisional control are facilitators of organizational learning and change, overcoming barriers such as established routines and political barriers. Participation may also facilitate learning, in turn leading to increased likelihood of longer-term changes in behavior. These attributes may also facilitate the successfully implementation of interventions to improve healthcare delivery. In a PAR/RCT approach, a 'joint' leadership structure with both a study and a site PI with local decision-making authority over choosing participants and intervention implementation may create a mixture of internal and external control that leads to more effective interventions.

There may also be benefit to integrating the ability to modify the intervention plan into the research design by building reflection into the intervention. Interventions that explicitly allow participants to reflect and respond to incremental changes in the outcome variables during the course of the intervention period may allow for adaptation of the intervention in ways that may make the intervention more effective. Creating opportunities for reflection within and across sites with a focus on sharing experiences may also allow interventions to evolve in more effective directions. These adaptations and their impact are important to understand. Rather than undermining the ability to generalize from results, a greater understanding of how local contexts and biases influence interventions may actually lead to findings that improve the ability of subsequent settings to implement the intervention. An example of such a strategy may include result feedback during specific ranges of time, such as sharing the impact of an intervention on process or patient outcomes.

To integrate PAR and RCT, new approaches to defining endpoints and their measurement will be required. In addition to the clinical endpoints that relate to the disease or population in question, endpoints chosen by local participants to help them monitor their progress should be added. Instead of pre-defined time periods at which endpoints are measured, the process of reflecting on the impact of an intervention in the clinical setting should become continuous, and the time it takes to implement an intervention may become an endpoint. This will allow for feedback that will help to strengthen the intervention, and will lead to a greater understanding of how the implementation process unfolds in each clinical setting. This understanding will be key to our ability to implement interventions successfully in other clinical settings. Thus, a greater appreciation of the process of intervention is a key lesson that must be derived from intervention studies.

An example of a PAR/RCT approach could include a multi-site study, half of which are randomized to an organizational intervention. The study team would partner with members of each intervention site to identify local barriers and create strategies to implement the intervention in a way that is deemed most effective by site participants. The intervention itself could include cyclical reflection exercises in which each site reviews results and modifies the intervention based on the results. In addition to these site-specific reflections, the intervention may also include times for all intervention sites to transfer ideas across sites. The timing of these reflective cycles and the timing of endpoint measurement could be modified based on these discussions. As part of the analysis of the results of the study, the themes of the reflections would be analyzed. An examination of any changes that might have occurred in control sites as a result of study participation would also be performed.

## Why including PAR may improve our ability to design more effective interventions and improve patient outcomes

At first glance, the suggestion to integrate RCT and PAR approaches may seem contradictory -- the former attempts to implement standardized interventions in an effort to reduce bias and increase generalizeability, while the latter is concerned with an individual system and its unique needs, rejecting the idea of the 'external researcher'. However, implementation research always occurs in the context of an organization, and for our efforts to become successful, new methodologies and approaches that recognize and respect each organization's unique characteristics, but still allow for a more universal understanding to be gained, must be developed. Rather than using standardized approaches to reduce bias, being explicit about differences and their impacts that will allow us to better understand the process of implementation, and it is this understanding that will lead to more successful implementation strategies. We suggest that an approach that builds on and integrates the RCT and PAR characteristics is more likely to advance our efforts than either approach alone.

The addition of elements of PAR to interventional research studies may be a way to better meet the needs of implementation research -- to meet the needs of generalizability while respecting local conditions that are important in individual healthcare settings. Additionally, these elements are well-suited to specific aspects of healthcare systems that reflect their complexity -- the role of relationships among healthcare workers, managers, and patients in potentially unpredictable settings. Incorporating PAR principles may provide us with a deeper understanding of healthcare systems and what is needed to improve them, as well as a better theoretical understanding of interventions and why they might be more or less effective in certain contexts. The results of implementation studies utilizing a practice facilitation approach suggests support for this approach, as practice facilitation focuses on improving relationships and communication within healthcare organizations.

Additionally, the explicit inclusion of reflection and 'sense making' is an important component of the PAR methodology that is critical for understanding CAS, where unanticipated or unexpected results of interventions may occur. The process of looking critically at the impact of an intervention and adapting to this impact may lead to more effective interventions. The application of sense making to organizations outside of healthcare supports this idea.

The approach of adapting elements of PAR to RCTs may seem problematic to both the strict adherents of both PAR, and to those of RCTs. For the former, the attempt to fit an approach that is meant to focus exclusively on the needs of participants into an intervention that is on some level superimposed may seem to negate the very principles of PAR. For the latter, the incorporation of this degree of latitude into an intervention may seem to nullify the purpose of performing an RCT, and the ability to generalize from its results.

We believe that these criticisms miss an essential point of this approach -- that organizations are dynamic, and that a greater understanding of the diverse processes through which general strategies may be implemented successfully is critical to implementation research. The question is not whether a diabetes registry or a clinical reminder applied in a specific way can lead to predictably improved outcomes for diabetic patients in six months; the question is whether these approaches applied uniquely in the contexts of individual healthcare systems are more likely to change these systems in sustained ways that will lead to improved outcomes. A key issue is whether an intervention is more or less likely to help to change the interconnections between elements of the system in a way that will lead to improved care. We can gain an understanding of whether certain types of interventions can be utilized in a manner across individual clinical systems such that outcomes are likely to improve. Instead of focusing on whether interventions are faithfully applied, we can learn from the myriad ways that participants apply interventions in their own settings, and from the degrees of change in outcomes that result.

Incorporating PAR principles may make the task of interpreting results of implementation trials more challenging, as it may be more difficult to assess true improvement in the setting of evolving interventions in organizations over time. However, they may also make interventions better suited to long-term successes by enabling us to implement more lasting organizational changes through the adaptive participation of those individuals who are most involved in the local process of care.

## Competing interests

The authors declare that they have no competing interests.

## Authors' contributions

JAP conceived the manuscript, conducted the initial review of studies of participatory action research, and completed the first draft of the manuscript. LL performed additional literature review, contributed to the first draft of the manuscript, and completed significant revision as part of the peer-review process. HL performed additional literature review, contributed to the application of the CAS framework, and contributed to the revision of the manuscript. JH contributed to the conceptualization and first draft of the manuscript. RRM contributed to the initial development of the manuscript, the application of the CAS framework, and the revision of the manuscript. All authors read and approved the final manuscript.
